# Horizon Scanning in Cancer Genomics: How Advances in Genomic Medicine Will Change Cancer Care Over the Next Decade

**DOI:** 10.1007/s40142-021-00200-7

**Published:** 2021-07-15

**Authors:** Lydia M. Seed

**Affiliations:** School of Clinical Medicine, University of Cambridge, Cambridge Biomedical Campus, Hills Road, Cambridge, CB2 0SP UK

**Keywords:** Cancer genomics, Oncogenomics, Cancer care pathway, Future of medicine, Cancer treatment, NHS, Genomic Medicine Service

## Abstract

**Purpose of Review:**

Advances in genomic medicine have the potential to revolutionise cancer patient care by driving forwards the clinical practice of precision oncology. This review aims to outline how genomic medicine advances may alter the care of cancer patients and their families over the next 10 years.

**Recent Findings:**

The translation of oncogenomic advances into the clinical environment will likely be facilitated by the increasing availability of next-generation sequencing technologies and the increasing genomic literacy of healthcare professionals. The implementation of the centralised, nationwide NHS Genomic Medicine Service promises to improve equity of cancer care and to facilitate personalisation of almost every stage of the care pathway, from informing population screening and how we diagnose cancer to delivering prognoses and surveillance. Advances in cancer pharmacogenomics, and other “omics” technologies, have a tremendous potential to optimise patient care. Genomic medicine advances will also enhance the care offered to cancer patients’ families.

**Summary:**

Genomic medicine advances are likely to transform almost every aspect of a cancer patient’s care pathway. Cancer care will profoundly improve over the next decade, increasing UK cancer survival rates and improving patient outcomes.

## Introduction

**Fig. 1:**
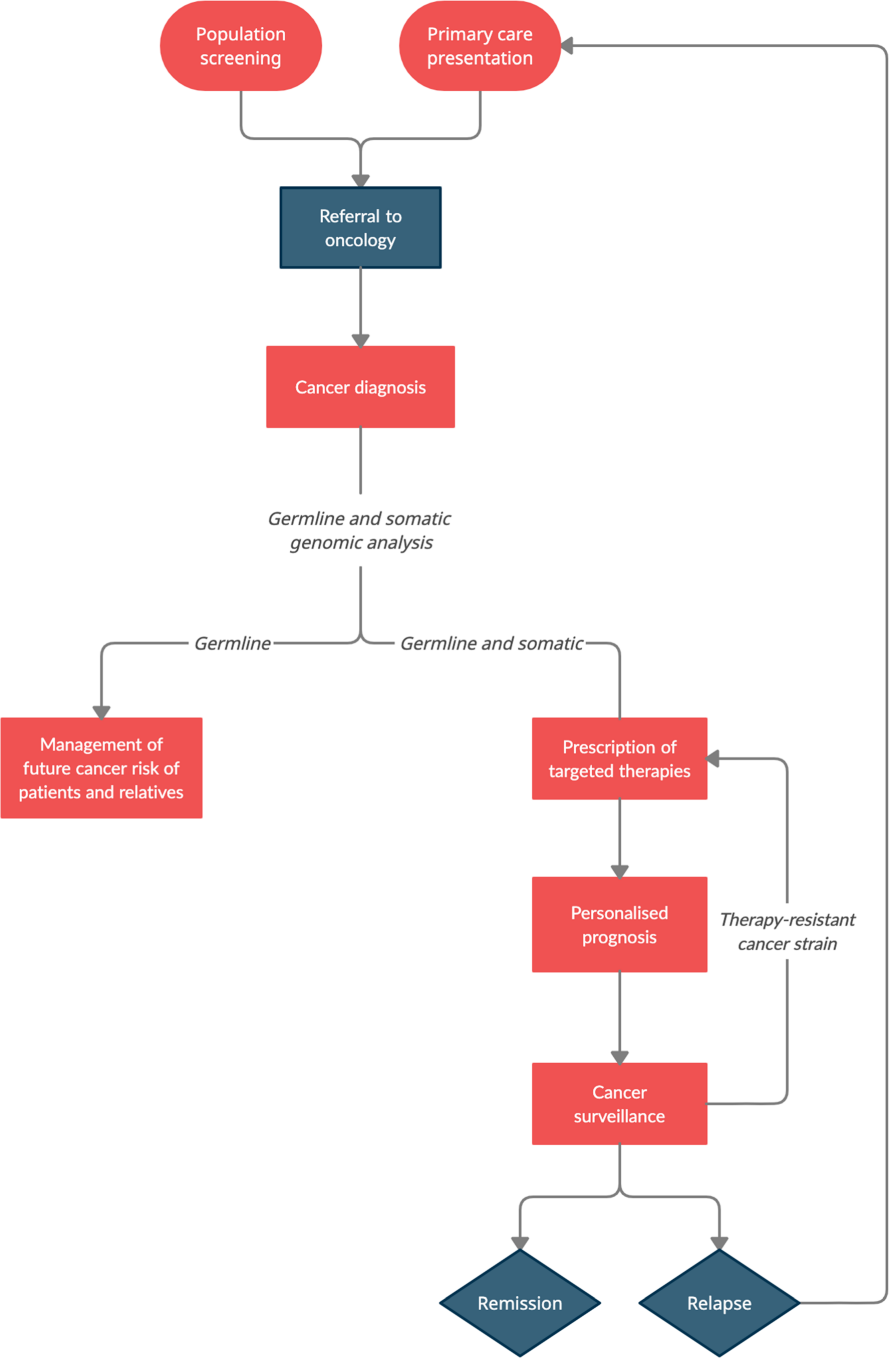
The future of precision oncology. A flowchart depicting the stages of a cancer care pathway: red boxes represent stages that will likely be personalised due to the clinical application of advances in genomic medicine within the the next 10 years; blue boxes represent the other stages

Less than a generation ago, a cancer diagnosis was a “death sentence”. Over the last decade, the incidence of cancer in the UK has increased by 5% [1]. However, the prognosis for the field of cancer care, and for those patients and their families who will depend upon it a decade from now, is bright. Advances in genomic medicine will drive forwards the clinical practice of precision oncology and have the potential to revolutionise and personalise almost every aspect of cancer care (Figure 1).

## Driving Factors

The translation of oncogenomic advances into the clinical environment will likely be driven by two key factors: the increased availability of next-generation sequencing (NGS) technologies and the enhanced genomic literacy of healthcare professionals.

### Increased Availability of Next-Generation Sequencing Technologies

With the advent of NGS technologies just over 10 years ago, the cost of whole genome sequencing (WGS) has rapidly reduced [2], facilitating its widespread availability and consequent application to basic and clinical cancer research and cancer patient care. The increased affordability of WGS enabled Genomics England, in partnership with NHS England, to deliver the 100,000 Genomes Project which sequenced both the germline and tumour genomes of over 15,000 cancer patients in the UK [3] and returned clinically actionable findings for many participants and their families. Moreover, the 100,000 Genomes Project now constitutes an invaluable global research resource, enabling the return of clinically actionable results for more participants as well as future patients and their families. Following its completion, NHS England established the NHS Genomic Medicine Service to deliver genetic testing to patients with rare disease and cancer, providing a direct pathway for advances in oncogenomics to reach future patients.

### Enhanced Genomic Literacy of Healthcare Professionals

In their 2020 “Strategic vision for improving human health at The Forefront of Genomics”, the National Human Genome Research Institute stated that “to fully realize the benefits of genomic advances” [4•], it is essential to enhance genomic literacy among healthcare professionals. In the UK in 2019, the recommendations of The Topol Review supported the aims of the NHS Long Term Plan, which includes the upskilling of the NHS workforce to promote the translation of genomic advances into the clinical environment [5]. Over the last decade, several educational initiatives have been launched to enhance the genomic literacy of the existing and future NHS workforce: Health Education England established the Genomics Education Programme to deliver “genomics education, training and experience for the healthcare workforce” [6]; and the General Medical Council stipulated in their Outcomes for Graduates that “newly qualified doctors must be able to apply... knowledge relating to... genetics, genomics and personalised medicine” to medical practice and integrate this into patient care [7].

## Equity of Care

Throughout the past decade, the effect of the “inexcusable” (Macmillan [8]) and “unacceptable” (Cancer Research UK [8]) postcode lottery on cancer patient survival has scandalised the media and general public with, according to Juliet Bouverie, Director of Services and Influencing at Macmillan Cancer Support, “6,000 people dying needlessly within 12 months of being diagnosed with cancer every year” [9]. However, the NHS Genomic Medicine Service, a network of local Genomic Laboratory Hubs underpinned by a single National Genomic Testing directory, will centralise oncogenomic services. Consistent and equitable oncogenomic care is in our sights, and, given that oncogenomics will influence almost every aspect of patient care, we can surely hope that within 10 years’ time, all patients diagnosed with the same cancer will receive comparable care, irrespective of postcode.

## Population Screening

In 2014, nearly half of all cancers were diagnosed at a late stage [10] and hence associated with poorer prognoses and patient outcomes. Advances in population screening promise to improve these aspects of cancer care.

### Genetic Screening

Our understanding of the genetic architecture of cancer susceptibility has greatly expanded over the last few decades. The high prevalence and mortality rate of some cancers, coupled with the availability of effective preventive interventions, signify that the time is upon us to utilise genomic risk factors to personalise national cancer screening and prevention programmes and to stratify the population to optimise early detection.

One approach will be to identify carriers of high-risk variants in cancer susceptibility genes, such as *BRCA1*, *BRCA2*, *MLH1* and *MSH2* [11•]. Currently in the UK, fewer than 10% of pathogenic *BRCA* variant carriers have been identified [12], despite the progressively lower threshold for testing based on family history and the expansion of cascade screening. Population risk profiling for breast, ovarian and colorectal cancers would be highly clinically valuable: frequency-penetrance profiles of pathogenic variants in *BRCA1*, *BRCA2*, *MLH1* and *MSH2* with breast, ovarian and colorectal cancers, respectively, are strong [13, 14]; breast and colorectal cancers are common and account for a large proportion of cancer deaths in the UK [1, 15]; and effective risk-reducing measures, such as screening, chemoprevention and surgery, are widely available for all three cancers [16, 17]. Furthermore, there is increasing evidence demonstrating that population screening for pathogenic variants in *BRCA1*, *BRCA2*, *MLH1* and *MSH2* would be highly cost-effective [18]. Since both clinical and health-economic evidence implicate population-wide testing to identify carriers of pathogenic variants in these genes, the implementation of such a programme is anticipated.

A vast number of polymorphisms that influence cancer risk have been identified through genome-wide association studies (GWAS) and can be combined into polygenic risk scores (PRS). The potential clinical utility of PRS-informed screening for many different cancers has been well-described [19, 20]: incorporating PRS with conventional risk factors improves overall risk predictions and stratification [21•]; and high polygenic risk explains 4.0–30.3% of cases, often exceeding the estimated contributions of modifiable risk factors and family history [21]. Integrating PRS with other clinical risk factors can personalise risk calculations and modify screening recommendations. For example, NICE currently recommends triennial mammograms for most women over 50 years of age [22]; however, a multi-factor risk-based model that includes PRS would identify 16.1% of women with a greater 10-year risk of breast cancer than the average 50-year-old, implicating earlier screening [23•]. Conversely, 32.0% of women would be identified as having a lower breast cancer risk at age 50, and delayed screening, to reduce the risk of harm from false positive, could be considered [23]. Trials investigating the benefit of integrating PRS to determine screening eligibility are currently ongoing [24], and their results may implicate population-wide PRS calculations to personalise the national breast cancer screening programme.

However, PRS calculated from the results of GWAS with participants from one ancestral group cannot be reliably transferred to populations of different ancestries [25]. Given that the majority of GWAS has been performed using participants of European ancestry, inequitable access to PRS-informed aspects of healthcare is of major concern. With the increasing discussions surrounding systemic racism and decolonising healthcare, it is hoped that these efforts will be reflected in future genomics studies so that powerful tools, such as PRS, can be made clinically available to all members of our diverse population.

Over the next 10 years, several key advances will likely encourage the clinical application of PRS to population screening for many different cancer types. Not only will more GWAS be performed to investigate the polygenic component of more cancers, but an increasing number will employ WGS, thereby eliminating the imputation stage and enabling detection of rarer variants that may influence cancer risk. Furthermore, GWAS will recruit larger numbers of participants, increasing the power of polymorphism-cancer associations. These advances will improve the accuracy and hence the potential clinical utility of PRS. In addition, the extremely low cost of microarrays would facilitate the implementation of population-wide genotyping to determine individuals’ PRS for different cancers.

### Asymptomatic Screening

Promising progress has been made in developing liquid biopsies that detect tumour-specific genomic biomarkers, such as circulating tumour DNA (ctDNA). The sensitivity of technologies to detect ctDNA in asymptomatic patients with early-stage tumours, which typically release lower levels of ctDNA, has hindered their clinical application as population screening tools. There are many unanswered questions pertaining to the utility of a ctDNA screening tool, such as whether it can detect a sufficient number of cancers at a treatable stage.

Later this year, a pilot study of the Galleri blood test—an innovative blood test, developed by GRAIL, that can detect the early stages of more than 50 types of cancer—will be launched [26]. In this study, 140,000 asymptomatic participants aged 50–79 years will receive annual blood tests for 3 years [26]. The results, which are expected by 2023, could implicate the rolling out of this service to more individuals throughout the UK or the selection of high-risk cohorts. It is hoped that this test will help achieve the goals set in the NHS Long Term Plan for earlier diagnosis of most cancers by 2028 [26, 27].

## Diagnosing Cancer

How we diagnose cancer will likely change over the next decade—both in terms of the diagnostic tests performed and the classification of the diagnoses reached.

### Diagnostic Tests

In addition to their potential use as a population screening tool, liquid biopsies may be utilised as a diagnostic tool.

A liquid biopsy requires a simple blood test. For some patients presenting with late-stage disease, who are too unwell to tolerate a general anaesthetic and undergo tissue biopsy procedures, a liquid biopsy would facilitate a cancer diagnosis and the commencement of appropriate therapies.

Additionally, since blood tests are easily accessible, liquid biopsies may be requested by primary care physicians when patients initially clinically present with suspicious symptoms, speeding up the time taken to reach a diagnosis. Several upcoming studies will assess the potential clinical utility of liquid biopsies to detect ctDNA in symptomatic patients where a cancer diagnosis is suspected. In the GRAIL pilot study 25,000 people with possible cancer symptoms will be offered the Galleri test [26]. The PREVAIL-ctDNA pilot study will aim to detect ctDNA in patients presenting with suspected pancreatic, lung, bladder and colorectal cancers and gastrointestinal stromal tumours [28] and to demonstrate that replacing tissue biopsies with liquid biopsies can enable clinicians to stratify patients and personalise cancer treatment earlier in the care pathway.

In tertiary care, genomic biomarkers may inform diagnoses for more complex cases. Proof-of-concept studies have demonstrated the potential clinical utility of microRNAs to detect germ-cell tumours—microRNA levels detected in the cerebrospinal fluid of two patients with abnormal pituitary stalk thicknesses that were too small to biopsy, accurately discerned which patient had a malignant germ-cell tumour and which did not [29]. Further studies with larger sample sizes may implicate microRNA testing in some patients.

The outcomes of the GRAIL and PREVAIL pilot studies, and future trials, may demonstrate the clinical utility of liquid biopsies for detecting tumour-specific biomarkers, enhancing our future diagnostic abilities and becoming part of routine clinical practice, from primary to tertiary care.

### Cancer Classification

I predict that over the next decade a new system of cancer classification, primarily based on tumour mutational profile rather than histological findings, will be developed, providing more clinically relevant information for the practice of precision oncology. For example, the same mutational signatures that arise from passenger mutations during oncogenesis are present in a broad range of cancer types—signature 2 has been found in 22 cancer types and attributed to the activity of the AID/APOBEC family of cytidine deaminases [30•]—and could enable cancer diagnoses that reflect the aetiology in a tumour-agnostic manner. Incorporating the mutational profile of tumours into cancer diagnoses in this way, or identifying driver mutations, will enable earlier access to targeted therapies.

## Pharmacogenomics and Targeted Therapies

Over the past two decades, major advances in cancer pharmacogenomics have transformed patient care. The next decade will likely see further advances in pharmacogenomics, as well as in other “omics” technologies, which will translate into clinical practice and improve cancer patient care.

### Gold Standard Model of Personalised Oncology

WGS of the somatic and germline genomes of cancer patients enables clinicians to tailor treatment to the mutational profile of the tumour and deliver personalised medicine: identifying clinically actionable driver mutations, such as in *BRCA*-mutation positive ovarian cancer, may implicate certain existing targeted therapies, namely the PARP inhibitor Olaparib [31]; and identifying a high tumour mutation burden may implicate immunotherapy, for example, hypermutated breast cancers may be more susceptible to PD-1 inhibitors [32].

However, while the NHS Genomic Medicine Service can provide the infrastructure to deliver these genetic tests, a gold standard model of how to integrate genetic testing results into clinical care pathways is needed. The Personalised Breast Cancer Programme (PBCP) may provide this. The pilot study of the PBCP, in 2016, was the first time that NHS breast cancer patients were offered WGS as part of routine treatment [33]. The aim of the study was to not only advance research but also to directly benefit the patients who donated samples and were undergoing treatment—often genetic analysis of tumours takes too long to benefit the patients who donate samples. The PBCP demonstrated that it is possible to return genetic testing results within 12 weeks of sampling—on average, turnaround time was 6–9 weeks—thereby enabling clinicians to react and alter treatment to optimise patient care [34]. The success of the PBCP has facilitated the rolling out of this service to more breast cancer patients in other centres across the UK [34]. The PBCP promises a model of gold-standard personalised oncology and a template that can be adapted to other cancer types. Within the next 10 years, the turnaround time of genetic testing results will likely decrease, and we may even see some first-line therapies prescribed that are informed by the mutational profile of patients’ tumours.

### Clinical Trial Enrolment

Some driver mutations identified in patients are currently not clinically actionable with respect to licenced therapies. They could, however, still have implications in clinical management and in the research and clinical trial setting. The centralised NHS Genomic Medicine Service will enable swifter enrolment to nationwide clinical trials that will stratify patients based on the driver mutations present in their tumours. This may improve the outcomes for participating patients. Furthermore, the larger sample sizes of current and future nationwide trials will improve the statistical power of results, providing more robust clinical evidence for personalised medicine in oncology. This will support the clinical approval of these drugs, leading to greater availability and numbers of driver-mutation-targeting drugs within the next decade.

### Mutational Signatures

Driver mutations cannot be identified in all tumours. Mutational signatures may inform therapeutic decisions in 10 years’ time, thus harnessing the full power of WGS. Although not yet clinically validated, some mutational signatures show pharmacogenomic promise—signatures 6, 15, 20 and 26 are associated with defective DNA mismatch repair [30•], and therefore tumours with these signatures may be susceptible to checkpoint inhibitors. The same mutational signatures are present in a broad range of cancer types, and therefore their potential therapeutic yield is high.

### Immunotherapy

In 2013, cancer immunotherapy was *Science*’s “Breakthrough of the Year” [35]. One of the outstanding scientific achievements within this field was the development of chimeric antigen receptor (CAR) T-cell therapy. CAR T-cell therapy involves harvesting a patient’s T-cells, genetically editing them to recognise and attack cancer cells, expanding the population of the genetically edited T-cells and reintroducing them into the patient. Challenges to the clinical implementation of this therapy include mitigation of toxicity and quality control of genetically engineered T-cells. However, towards the end of the last decade, the NHS began to offer this revolutionary therapy to children and adults with certain haematological malignancies, such as specific subtypes of non-Hodgkin lymphoma [36]. At the start of this year, this was expanded to include certain patients with mantle cell lymphoma [37]. Over the next 10 years, this personalised medicine will likely continue to be offered to more patients with a broader range of cancers.

In recent years, blockade of the programmed death 1 (PD-1) pathway has emerged as a highly effective therapy for tumours with a high mutational burden. The PD-1 pathway is upregulated in many tumours, microenvironments and immune cells and, via negative feedback mechanisms, inactivates T-lymphocytes and contributes to blocking the “cancer immunity cycle” [38]. PD-1 pathway blockades have evoked a remarkable clinical response in patients with many different types of cancer [39, 40]. Results from a phase 3 clinical trial demonstrated that patients with high mutational burden colorectal cancer who received the anti-PD-1 monoclonal antibody pembrolizumab as a first line therapy had a significantly longer progression-free survival and fewer treatment-related adverse events than those who received standard chemotherapy [41]. Further, a growing body of evidence suggests that high mutational burden tumours are less responsive to conventional chemotherapy [42], which is the routine standard of care for high mutational burden colorectal cancers. It is hoped that in the coming years, PD-1 pathway blockades will become the standard first-line treatment for these patients and expanded to include more patients with high mutational burden tumours.

### Epigenetics

Cancer is not only a disease of the genome. In addition to genomic abnormalities, most cancers display aberrant epigenomic states, which may arise via several pathways: epigenetic priming of cancer-initiating cells [43]; somatic mutations in genes encoding proteins that regulate epigenetic modifications; and metabolite changes, such as accumulation of fumarate, which may drive genome-wide methylation changes [44]. These altered epigenetic states may drive oncogenesis, influence resistance to chemotherapy and resist immune invasion. The epigenome is therefore an attractive therapeutic target.

At the beginning of the last decade, the DNA methylation inhibitor azacitidine became a recommended treatment for haematological malignancies, such as acute myeloid leukaemia (AML) [45]. More recently there has been a surge in drug discovery efforts targeting the epigenome—small molecule inhibitors target gain-of-function mutations, and loss-of-function mutations can be targeted through synthetic lethality [46, 47]. Some epigenetic drugs can modulate the efficacy of immunotherapies, and epigenetic inhibitors have been shown to overcome drug resistance. Ongoing clinical trials of epigenetic therapies for many cancers—including a phase 3 clinical trial investigating the histone deacetylation inhibitor, entinostat, for breast cancer [48]—will conclude within the next decade and will likely prompt their clinical use, especially in combination with other therapies.

### Single-Cell RNA-Sequencing

Intratumour heterogeneity is a significant barrier to the effective treatment of cancer and is regarded as a major driver of resistance to therapy and metastasis [49]. The complex functional heterogeneity within tumours arises from the genetic heterogeneity among malignant cells derived from the genetic variation and simultaneous Darwinian selection of tumour cells that lead to their clonal expansion, the plethora of diverse cell types within the tumour microenvironment and the varied transcriptional responses of tumour cells to factors in the tumour microenvironment. Recently developed single-cell RNA-sequencing (scRNA-seq) technologies can finely characterise the transcriptomic architecture of tumours and their microenvironments. The generation of the Human Cell Atlas, a knowledgebase containing single-cell transcriptomes from normal and pathological samples that can act as a reference, has facilitated the precise cell-type annotation of tumour samples. Further, scRNA-seq does not require special infrastructure, and so an increasing number of research studies are employing scRNA-seq and demonstrating its potential to reveal therapeutic targets within cancer-microenvironment interactions across a wide range of cancers—from breast cancer [50] to metastatic lung adenocarcinoma [51] to muscle-invasive urothelial bladder cancer [52]. Precision oncology in the future will involve the functional characterisation of intratumour heterogeneity, resulting in improved patient outcomes. Within the next decade, it is likely that large-scale scRNA-seq cancer research studies and clinical trials will be conducted, and we may even see scRNA-seq emerging into clinical practice.

## Personalised Prognosis

The mutational profile of tumours can indicate cancer properties and disease progression. A Wellcome Sanger Institute research team has developed an algorithm to predict the outcomes of patients with AML. The algorithm incorporates the driver mutations present in the patient’s cancer with other clinical features, such as the patient’s age at diagnosis, their white cell count and the presence of splenomegaly [53•]. Although not yet validated for clinical use, it likely will be within the next decade, enabling oncologists to deliver personalised prognoses to AML patients. A similar model for myeloproliferative neoplasms is currently being developed, and over the next 10 years, personalised predictors for many other cancer types will likely be produced and clinically validated.

Personalised prognoses will inform palliative care decisions and enhance holistic cancer patient care. Often cancer patients and their families want to know how long they are expected to live [54]. Clinicians will be better equipped to answer the common question, “How long do I have left?” A personalised prediction of life expectancy can empower patients and their families to make more informed decisions surrounding end-of-life care and better plan their remaining time. Additionally, navigating cancer care can be intimidating for patients as a lot of medical jargon is used. A personalised prognosis is an easily accessible way of conveying information to a patient and their family about their illness. Personalised prognoses are a noteworthy example of how genomic medicine advances will enhance all aspects of the biopsychosocial model of medical practice.

## Surveillance

We will soon be able to offer patients personalised cancer surveillance. Non-invasive techniques employed in cancer detection may also be utilised in the monitoring of patients, with the potential to transform cancer patient care throughout the therapeutic pathway. Serial liquid biopsies will allow us to monitor patients’ responses to treatments and enable early detection of emerging therapy-resistant clones within the primary cancer and metastases. There are promising results from recent studies: patient-specific mutations in plasma ctDNA have been quantified to determine tumour burden, monitor response to therapy and identify relapses earlier in patients with metastatic breast cancer [55] and colorectal cancer [56]; and microRNAs have detected micro-metastases in paediatric malignant germ-cell tumours that are undetectable on CT scans [57].

## Family Screening

Over the next decade, our understanding of the inherited component of cancer risk will continue to expand. Firstly, the interpretation of germline variants in genes already associated with increased cancer risk will improve. Bioinformatics research and advances in computational power will facilitate the development of more advanced data analysis pipelines that can better functionally annotate and prioritise variants in the germline genome of cancer patients. Secondly, the wealth of oncogenomic “big data” available from the NHS Genomic Medicine Service, and other research consortia such as the COSMIC database [58], will enable an increasing number of novel cancer predisposition syndromes to be identified, improving the yield of germline genomic testing. Improved understanding of heritable cancer risk will enable us to offer cascade screening to more patients’ families and identify more at-risk relatives.

Identification of family members with an inherited increased cancer susceptibility will improve following implementation of recent international recommendations produced by the European Society of Medical Oncology. These guidelines outline a germline-focussed approach to somatic tumour sequence analysis and have refined the indications for germline follow-up testing [59]. This will optimise the detection of clinically useful, true germline variants and improve the yield of germline follow-up testing.

Furthermore, through the expanding NHS Genomic Medicine Service, access to genetic counselling will widen, and more cancer patients and their families will be offered this invaluable support. By the end of the next decade, an increasing number of cancer patients’ family members will be better equipped and supported as they navigate their genetic testing results to make genomic-informed decisions regarding implementing risk-reducing measures prophylactically.

## Challenges

In addition to challenges mentioned above, several other obstacles may hinder the application of genomic medicine advances to the clinical practice of oncology over the next 10 years.

While the cost of NGS technologies is now relatively low, the incidence of cancer is both high and increasing [1]. To provide oncogenomic services for ~367,000 new cancer patients every year in the UK will be costly [1]. Moreover, the cost of novel cancer therapies can be high, and so the threshold of improvement in patient outcomes that needs to be demonstrated before their clinical approval may be stringent. Additionally, the time taken for research studies and clinical trials to be conducted, their findings published, and the results translated into clinical practice can be lengthy. The impact of oncogenomic advances on cancer patient care may therefore be slower than desired, and many of the advances mentioned herein may only be emerging in clinical practice towards the end of the next decade.

## Conclusions

Advances in genomic medicine over the next 10 years promise to revolutionise the care of cancer patients. Critically, we have never needed personalised medicine in the field of oncology more. With the disruption, for over a year, to NHS cancer services subsequent to the COVID-19 pandemic, there is an anticipated cancer epidemic [60]. Urgent referrals have been missed, chemotherapy and surgical treatments have been halted, and the rate of cancer detection has been much lower [61]. We face an imminent influx of cancer patients with late-stage disease and poor prognoses, and there is a pressing need for the most effective, targeted therapies. Genomic medicine offers a great deal of hope in these dark times. As we continue to develop the knowledge and skills to practise precision medicine, we hope to deliver for these patients optimal personalised care. It is my hope that, despite the challenges we face from the ongoing pandemic, thanks to the promise of genomic medicine, we will achieve NHS England’s 2028 target for early diagnosis of 75% of all cancers [27], and that, over the next 10 years, we will see increased UK cancer survival rates.
